# Efficacy and Safety of Wenbu Zhibi Granule in Patients with Ankylosing Spondylitis: A Multicenter, Randomized, Double-blind, Placebo-controlled Trial

**DOI:** 10.1155/2021/8683600

**Published:** 2021-11-25

**Authors:** Helou Zhang, Yang Yu, Weibin Du, Fengqing Wu, Yang Zheng, Conglin Ren, Huateng Zhou, Yijiang Wu, Yang Gao, Weifan Ren, Renfu Quan

**Affiliations:** ^1^Zhejiang University of Traditional Chinese Medicine, 548 Binjiang Road, Hangzhou, Zhejiang, China; ^2^Institute of Spine, Shanghai University of Traditional Chinese Medicine, 725 South Wan-Ping Road, Shanghai, China; ^3^The Affiliated JiangNan Hospital of Zhejiang Chinese Medical University Orthopedics, 156 Yucai Road, Xiaoshan, Hangzhou, Zhejiang, China; ^4^Hangzhou Xiao Shan Hospital of Traditional Chinese Medicine, Hangzhou 312001, China; ^5^Research Institute of Orthopedics, the Affiliated Jiangnan Hospital of Zhejiang Chinese Medical University, Hangzhou 312001, China

## Abstract

*Background*. Ankylosing spondylitis (AS) is a chronic disease in which the column is the main lesion. It is caused by a combination of genetic and environmental factors, mainly involving the axial skeleton, resulting in column rigidity and difficulty in movement, and there may be different degrees of eye, lung, cardiovascular, kidney, and other organ damage. Long-term treatment lacks in ankylosing spondylitis. Wenbu Zhibi granule (WZG) is a prescription handed down from the history of Chinese medicine for thousands of years, which is used to treat the pain of patients with AS and to prevent the further development of the disease. However, there is no scientific evidence based on clinical trials to evaluate the efficacy and safety of WZG for ankylosing spondylitis. *Methods/Design*. We will conduct a multicenter, randomized, double-blind, placebo-controlled trial to evaluate the efficacy and safety of the WZG in the treatment of AS. We will randomly assign 100 patients with active AS to two groups, treated for 16 weeks. The primary efficacy endpoint is the proportion of subjects who reached 40% improvement criteria proposed by Assessment of SpondyloArthritis International Society (ASAS40) at 16 weeks from baseline, the secondary efficacy endpoint includes ASAS20 response rate, ASAS partial remission response rate, 5/6 improvement criteria proposed by ASAS (ASAS5/6) response rate, and change in the Spondyloarthritis Research Consortium of Canada (SPARCC) MRI spine score, Bath Ankylosing Spondylitis Disease Activity Index (BASDAI), Bath Ankylosing Spondylitis Functional Index (BASFI), Ankylosing Spondylitis Disease Activity Score (ASDAS), linear Bath Ankylosing Spondylitis Metrology Index (BASMI), ankylosing spondylitis quality of life (ASQoL). In addition, the time points will be set as baseline, 2 weeks, 4 weeks, 8 weeks, 12 weeks, 16 weeks, 24 weeks, and 48 weeks. *Discussion*. The results of this study will elucidate the efficacy and safety of WZG and provide an appropriate treatment option for patients with AS. Trial registration: ClinicalTrials.gov ID: https://clinicaltrials.gov/ct2/show/ChiCTR2000041010. (Chinese Clinical Trail Registry, Registered 16 December 2020, http://www.chictr.org.cn).

## 1. Introduction

Ankylosing spondylitis (AS) is a chronic progressive inflammatory rheumatism which is usually present in early adulthood and strongly associated with the HLA-B27 gene with a prevalence of about 1.4% [1]. AS, otherwise known as radiographic axial spondyloarthritis, is a type of spondylitis, which also includes psoriatic arthritis, arthritis associated with inflammatory bowel disease, and reactive arthritis [2]. Each of these spondylitis is progressive and can develop into a typical ankylosing spondylitis. AS, characterized by chronic back pain and stiffness of the pelvis and lower back, mainly affects the axial skeleton such as the column and sacroiliac joint [3]. Extra-articular manifestations in AS include acute uveitis, peripheral arthritis, enthesitis (inflammation of where tendons insert on bones), psoriasis, aortic root, and gut inflammation [4]. AS is a hidden onset and long course of disease. With the development of the disease, it eventually causes joint fibrosis, ankylosis, loss of joint function and a high rate of disability [5].

According to the existing international management recommendations [6, 7], long-term drug and nondrug therapy is recommended to control the disease. The main drug treatments are nonsteroidal anti-inflammatory drugs (NSAIDs), antirheumatic drugs (DMARDs), and biological DMARDs (TNF *α* blocker or IL-17 blocker). Some studies have shown that the long-term use of NSAIDS does not lead to substantial improvement in the treatment of the disease but also increases the risk of gastrointestinal and cardiovascular diseases. [8] DMARDs such as methotrexate and sulfasalazine are just limited to the improvement of muscle stiffness and discomfort, but it cannot avoid the occurrence and progression of AS. [2] Poddubnyy found that long-term use of TNF *α* slowed the radiographic progress of patients with AS, but the effectiveness was limited to remission rather than radical cure. [9] Whether IL-17 blockade might reduce the progression of new bone formation is unknown [10], and up to 40% of patients do not respond to them. [11, 12] Therefore, on the basis of reducing inflammation, we aim to look for alternative therapies to relieve symptoms and improve the quality of life of the patients with AS. As a complementary and alternative medicine, some traditional Chinese medicines such as the WZG may have the potential to relieve AS symptoms and reduce disease activity. [13] The WZG is combined with two well known traditional Chinese folk medicine: the Duhuo Jisheng granule and Wutou granule. Pharmacological studies show that both of the two traditional granules can inhibit inflammatory factors, reduce degenerative lesions of articular cartilage, and promote articular cartilage regeneration. [14–17].

Although the WZG have been used clinically in our hospital for decades, the efficacy and safety of the WZG still need evidence-based medical research. Hence, we plan to conduct a randomized, double-blind, placebo-controlled trial to confirm the efficacy and safety of the WZG in fighting against AS.

## 2. Methods

### 2.1. Participants

This study will enroll patients who are signed up by website, posters, and telephone from three hospitals (Zhejiang Provincial Hospital of Traditional Chinese Medicine, Department of Obstetrics and Gynecology, Zhongshan Hospital of Zhejiang Province and the Affiliated JiangNan Hospital of Zhejiang Chinese Medical University orthopedics). The participants who sign the informed consent form will be divided into the test group and control group according to the random number method and will be followed up for 48 weeks.

### 2.2. Inclusion and Exclusion Criteria

Participants meeting the following requirements will be included and excluded, as shown in Table 1.

### 2.3. Study Design

**Figure 1:**
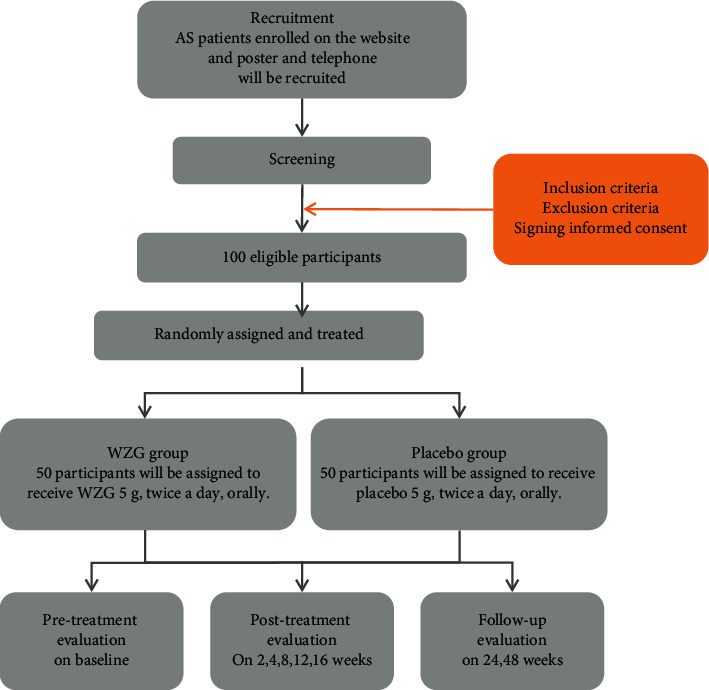
Project overview. AS: ankylosing spondylitis; WZG: Wenbu Zhibi granule.

This is a multicenter, randomized, double-blind, parallel-group, placebo-controlled clinical trial (Figure 1),.

Which is being conducted at three centers in Zhejiang, China: Zhejiang Provincial Hospital of Traditional Chinese Medicine, Department of Obstetrics and Gynecology, Zhongshan Hospital of Zhejiang Province and Affiliated JiangNan Hospital of Zhejiang Chinese Medical University orthopedics to evaluate the efficacy and safety of the WZG in the treatment of AS.

### 2.4. Sample Size Calculation

According to our primary study of the WZG from January 2020 to August 2020, the response rate of ASAS40 was 84.5% in the WZG group and 54.4% in the WZG placebo group. We plan to provide at least 90% power and a (two-sided) 5% significance level for detecting treatment differences. [19] When an assignment ratio of the groups of 1 : 1 are applied, according to the formula of sample size calculation:$N_{1}=N_{2}={\left[u_{\alpha /2}\sqrt{2\bar{p}\left(1-\bar{p}\right)}+u_{\beta }\sqrt{p_{1}\left(1-p_{1}\right)+p_{2}\left(1-p_{2}\right)}/p_{1}-p_{2}\right]}^{2}$ (*N*_1_,*N*_2_ are the size of each group; *p*_1_ is the ASAS40 response rate of WZG group and *p*_2_ is the rate of WZG placebo group; $\bar{p}$ is the mean of *p*_1_ and *p*_2_; *u*_*α*/2_ = 1.96 when type I error is 0.05; *u*_*β*_ = 1.282 when type II error is 0.1 in two-sided tests), accounting for a dropout rate of 10%, at least 50 subjects per group are required, for total of 100 subjects.

### 2.5. Randomization and Masking

The random assignment codes will be generated on the computer by the statistical professionals using SAS software, and the project team will assign a special person independent of this study to keep the group information confidential. According to the randomized sequence, a random distribution sheet with two copies shall be made and bound into a book to make a random distribution book with cover and instructions. One of the couplets on the top is to collect the enroll information, and the other is to show the allocation information. The serial numbers of the couplet around the sealant are the same and leave blank at the same areas of the couplet for signing the enter information. The content of the top couplet can be completely copied to the bottom one. To avoid exposing the allocation information in advance, the back of the bottom couplet should be black-printed. When the subjects are sure qualified, the researchers selected the corresponding couplet in a sequential order. Exposing the allocation information in the bottom couplet, and the subjects will be allocated to the group designated on the bottom couplet. This random allocation book will be printed by the professional printing service. All investigators except the special person will not know the corresponding relations between sequence numbers and different groups until the trial are completed.

### 2.6. Blinding

None of the researchers will contact the special person or the pharmaceutical company or the printing service in this trial. The staff of the pharmaceutical factory and printing service will not be involved in other parts of the study. The special person will be separated from all researchers. Therefore, participants, doctors, nurses, researchers, and statisticians (analyzing data) have no access to the study information and will not know the relationship between the numbers and groups until the end of this trial.

### 2.7. Medication

The WZG will be produced, packaged, and marked by the factory in Zhejiang Province.  Table 2 lists the components of the WZG.

The WZG, using the improved spray drying granulation method, [20] is prepared as follows:

#### 2.7.1. Extraction

The herbs are placed in a ceramic pot, then pour 1,000 liters of distilled water into the pot to soak the materials for 1 hour, then boil it at 100°C for 1 hour for the first extraction; pour the liquid extract into another pot, and add 1000 liters of distilled water and boil it at 100°C for 1 hour to extract it again; and then repeat it for the third extraction.

#### 2.7.2. Concentration

Mix the liquid that is collected and concentrate it at 60°C (660 mmHg) with a 1 : 1.30 concentration ratio (80°C). Then spray-dry it into powders before crushed and sieved through a mesh size of 80.

#### 2.7.3. Packing

Finally, the granules are packed (5 g per bag) and stored in a clean and dry room. The WZG placebo is consisted of the WZG extract (10%) and bitters (90%). Some food additives will be added to make the taste, color, smell, and shape of placebo similar to the WZG.

### 2.8. Allocation and Intervention

All patients are randomly divided into the WZG group (experimental group) and placebo group (control group). Patients either take oral WZG 5 g (one pack, dissolved in 200 mg of hot water) twice a day or matching placebo for a 16-week period.

Two groups of supportive therapy: one type of NSAIDs, DMARDs, or biological DMARDs can be used during the observation period, such as methotrexate. In addition, patients should provide a detail list of medication usage during the trial.

Study visits occurred at baseline and at weeks 2, 4, 8, 12, 16, 24 and 48. The schedule of this trial is shown in Table 3.

## 3. Outcome Measures

### 3.1. Primary Measurement

The main outcome measure is the ASAS40 response in patients. The ASAS40 response has been used as a primary endpoint in clinical trials of patients with AS. [21] The ASAS40 response criteria is at least 40% improvement and an absolute improvement of at least two units on a numerical rating scale of 0–10 from baseline in at least three of the following four domains, with no worsening in the remaining domain: (1) patient global assessment of disease activity; (2) patient assessment of back pain; (3) the Bath Ankylosing Spondylitis Functional Index (BASFI); (4) inflammation: the mean of the BASDAI questions on severity and duration of morning stiffness.

Similarly, ASAS20 response (at least 20% improvement and and at least 1 unit of absolute change, with no worsening of a similar amount in the fourth domain), ASAS5/6 (at least 20% improvement in 5 of 6 domains—the same 4 domains as the ASAS40 response criteria plus 2 extra domains, acute‐phase reactants and spinal mobility), and ASAS partial remission (Assessment of low disease activity state and remission, a value of <2 on a 0–10 scale in each of the 4 ASAS40 domains) are also validated measure to assess signs and symptoms, but the advantage of the ASAS40 response criteria set is simplicity: it is based on the same domains as those for the ASAS20 response criteria with no qualitative distinction. [22].

### 3.2. Secondary Measurements

The secondary endpoint includes the proportion of patients who achieve the ASAS20, ASAS5/6, ASAS partial remission, and change from baseline to weeks 2, 4, 8, 12, 16, 24, and 48 in the following outcomes: the Spondyloarthritis Research Consortium of Canada (SPARCC) MRI spine score, Bath Ankylosing Spondylitis Functional Index (BASFI), Bath Ankylosing Spondylitis Disease Activity Index (BASDAI), Ankylosing Spondylitis Disease Activity Score (ASDAS), Bath Ankylosing Spondylitis Metrology Index (BASMI), and Ankylosing Spondylitis Quality of Life (ASQoL). Unlike ASAS40, the secondary measurements may be used to monitor the actual level of disease activity to define a state of remission or low disease activity and to measure response to treatment. [23].

  SPARCC MRI spine score: Within clinical trials, MRI is often repeated over short periods to test the efficacy of treatment. The ˀSpondyloarthritis Research Consortium of Canada (SPARCC) MRI spine score, the assessment of structural damage, is frequently used because it is a feasible, reproducible, and responsive method for measuring spinal inflammation on a continuous scale with good sensitivity to change. [24–26].
  BASFI: The Bath Ankylosing Spondylitis Functional Index (BASFI) is used to define and monitor physical functioning in patients with AS. It is composed of 8 items concerning activities referring to the functional anatomy (bending, reaching, changing position, standing, turning, and climbing steps) and two items assessing the patients' ability to cope with everyday life with a response scale (0–10) or visual analog scale (0–10 cm) anchored by “easy” and “impossible.” [27].
  BASDAI: Historically, the Bath Ankylosing Spondylitis Disease Activity Index (BASDAI) has been the most widely used and comprehensive self-administered measure of disease activity in AS. The BASDAI is user friendly, reliable, and sensitive to change and reflects the entire spectrum of disease. [28] It is a combined disease activity score, ranging from 0 (no disease activity) to 10 (maximal disease activity), including patient-reported levels of back pain, fatigue, peripheral joint pain and swelling, localized tenderness, and the duration and severity of morning stiffness. A cut off of 4 is used to define active disease. [29].
  ASDAS: The Ankylosing Spondylitis Disease Activity Score (ASDAS) has been used to assess treatment outcomes in clinical trials and to monitor disease activity in patients with AS. [30] Unlike BASDAI, ASDAS is a composite disease activity instrument which incorporates both objective inflammatory markers such as C reactive protein (CRP), the erythrocyte sedimentation rate (ESR), and patient-oriented measures (back pain, duration of morning stiffness, patient global assessment, and peripheral joint pain). [31] Its response option is a continuous scale from zero with no defined upper end determined by the level of the CRP or ESR.
  BASMI: The Bath Ankylosing Spondylitis Metrology Index (BASMI) can quantify the mobility of the axial skeleton in AS patients and allow objective assessment of clinically significant changes in spinal movement. It contains clinical measures of cervical rotation, tragus to wall distance, lumbar flexion, lumbar side flexion, and intermalleolar distance with a score from 0 to 10 based on individually defined cut points. Ranges are given as cervical rotation (>85.0° to ≤8.5°), tragus to wall (<10 cm to ≥38 cm), lumbar flexion (>7.0 cm to ≤0.7 cm), lumbar side flexion (>20.0 cm to <1.2 cm), and intermalleolar distance (≥120 cm to <30 cm). [32].
  ASQoL: The Ankylosing Spondylitis Quality of Life Scale (ASQoL) is used to measure the impact of AS on health‐related quality of life from the patient's perspective. The questionnaire includes items related to the impact of disease on sleep, mood, motivation, coping, activities of daily living, independence, relationships, and social life with 0 scored for a “no” and 1 scored for a “yes” for each item. The total score is the sum of the individual responses. The score range is 0–18, with higher scores reflecting greater impairment of health‐related quality of life. [33].

### 3.3. Safety Assessments

All patients receiving treatment will be evaluated for safety of the treatment. The vital signs (body temperature, pulse, respiration, heart rate, and blood pressure) of the patients will be collected at each visit. Data for adverse events (AE) defined as began or worsened in severity after the first dose of treatment through 30 days after the last dose will be recorded during the research. Laboratory tests will be conducted at baseline and week-16, including blood routine, urine routine, feces routine, liver function, and kidney function including the levels of HGB, PLT, AST, ALT, BUN, and CRE.

### 3.4. Statistical Analysis

SPSS22.0 statistical software will be used for statistical analysis. The continuous variables will be expressed as mean ± standard deviation, and the categorical data will be presented as percentage using the chi-square test or Fisher's exact test. The *T* test and nonparametric tests will be used to compare the differences between groups, and repeated measures analysis of variance will be used to analyze the data in different time points. All hypothesis tests will use a two-sided test, and *P* < 0.05 is statistically significant.

### 3.5. Data Collection and Management

All the data will be collected, and all sensitive information will be preserved in the Affiliated JiangNan Hospital of Zhejiang Chinese Medical University orthopedics. Epidata (version 3.0) procedure will be used to restrict data values. Two independent investigators will compare and double-check the data to rule out the difference based on the source documents of this trial.

### 3.6. Quality Control

To maintain the quality of this trial, the Affiliated JiangNan Hospital of Zhejiang Chinese Medical University orthopedics will monitor the study documents and procedure and be responsible for quality control.

## 4. Discussion

AS, otherwise known as radiographic axial spondyloarthritis as the lesions of the sacroiliac joint or column, could be observed in the radiographic image. [2] AS is characterized by inflammation with an excess spinal bone formation on the axial skeleton which could result in progressive and irreversible structural damage of bones [34, 35] and therefore, cause stiffness, pain, and reduced functions among patients. [5, 6] The main objectives of AS treatment are maximizing long-term healthy quality of life by controlling symptoms and inflammation, preventing progressive structural damage, and maintaining or normalizing functional and social participation. [36, 37] Treatment with NSAIDs alone is often insufficient to control the disease. AS/EULAR does not recommend DMARDs for axial arthropathy because the evidence of its ability to alter the natural course and imaging progression of AS is insufficient. [1, 2, 38] Owing to the immune system suppressed, infection is a major complication in patients with AS using some current biological DMARDs. [7, 39–42] Facing the difficult problem of treating AS, it is a great clinical significance to find new drugs for treating AS, and it may bring good news to the patients with AS.

Treatment of traditional Chinese medicine in AS has accumulated rich experience in clinical. The WZG is a granule-shaped herbal medicine combined with two classical prescriptions: the Duhuo Jisheng granule and Wutou granule. The Duhuo Jisheng granule could nourish Xiajiao to prevent the further development of the disease, and the Wutou granule could nourish Zhongjiao to alleviate the pain of patients with AS. Both of those granules are used as conventional prescriptions for thousands of years to treat patients with AS. Its effectiveness has already been experienced, but there is no clear and convincing evidence to be scientifically confirmed. Therefore, we designed this randomized, double-blind, placebo-controlled clinical study, on the basis of our initial trial, to scientifically prove the traditional prescription's effectiveness and safety.

In conclusion, the WZG is a new compound traditional Chinese medicine, and this study is built on our preliminary open trial with a small sample, whereas some clinical effects have been observed in the early stage, it cannot replace the first-line drugs in the treatment of AS completely. The multicenter is only located in Zhejiang Province, which leads to the regional limitation of the patient source. Despite the limitations, the results of this study will elucidate the efficacy and safety of the WZG and provide an appropriate treatment option for patients with AS.

## Figures and Tables

**Table 1:** Screening criteria of participants.

Inclusion criteria	Exclusion criteria
Diagnosed with AS according to the revised New York standards [18]	Total spinal ankylosis
Having active disease indicated by BASDAI score ≥4	Other rheumatic diseases such as SLE, RA
Older than 18 and the age of first onset was less than 45	Other serious diseases of vital organs
Consenting to the use of effective contraception during the trial period	Pregnancy or lactation
Voluntary participating in this study, adopting by the ethics committee, ensuring the test compliance and signing the informed consent	Cancer, cognitive, or mental disorders
	Suspected or confirmed history of alcohol abuse
	Infection and severe allergic reactions
	Active systemic infection within 2 weeks before the baseline visit
	Abnormal laboratory indexes of liver and kidney function

AS: ankylosing spondylitis; BASDAI: bath ankylosing spondylitis disease activity index; SLE: systemic lupus erythematosus; RA: rheumatoid arthritis.

**Table 2:** Components of the Wenbu Zhibi granule.

Chinese name	Latin name	Proportion (kg)
Du Huo	radix angelicae pubescentis	9
Sang Jisheng	radix loranthi seu visci	6
Du Zhong	coratex eucommiae ulmoidis	6
Niu Xi	radix achyranthis bidentatae	6
Xi Xin	herba asari cum radice	3
Qin Jiao	radix gentianae macrophyllae	6
Fu Lin	sclerotium poriae cocos	6
Rou Guixin	radix cinnamomi cassiae	6
Fang Feng	radix ledebouriellae divaricatae	6
Chuan Xiong	radix ligustici wallichii	6
Ren Shen	radix panacis ginseng	6
Gan Cao	radix glycyrrhizae	6
Dang Gui	Radix angelicae sinensis	6
Shao Yao	radix dioscoreae oppositae	6
Sheng Dihuang	radix rehmanniae	6
Ma Huang	Herba ephedrae	9
Huang Qi	Radix astragali	9
Chuan Wu	Radix aconiti kusnezoffii	6

**Table 3:** Schedule of the measures.

Measure time point study period	Per-treatment period		Treatment period					Follow-up period	
	−2 week	Week 0	Week 2	Week 4	Week 8	Week 12	Week 16	Week 24	Week 48
*Enrollment*									
Inclusion criteria	√								
Exclusion criteria	√								
Informed consent	√								
Allocation		√							

*Interventions*									
WZG		√							
WZG placebo		√							

*Assessments*									
ASAS40		√	√	√	√	√	√	√	√
BASDAI	√	√	√	√	√	√	√	√	√
ASAS20		√	√	√	√	√	√	√	√
ASAS5/6		√	√	√	√	√	√	√	√
ASAS partial remission		√	√	√	√	√	√	√	√
SPARCC MRI spine score		√	√	√	√	√	√	√	√
BASFI		√	√	√	√	√	√	√	√
BASMI		√	√	√	√	√	√	√	√
ASDAS		√	√	√	√	√	√	√	√
ASQoL		√	√	√	√	√	√	√	√
ESR, CRP		√	√	√	√	√	√	√	√
Vital signs	√	√	√	√	√	√	√	√	√
Blood, urine, feces routine		√					√		
Liver and kidney function	√	√					√		
Compliance assessments			√	√	√	√	√	√	√
AE		√							

WZG: Wenbu Zhibi granule; BASDAI: bath ankylosing spondylitis disease activity index; SPARCC: spondyloarthritis research consortium of Canada; BASFI: bath ankylosing spondylitis functional index; BASMI: bath ankylosing spondylitis metrology index; ASDAS: ankylosing spondylitis disease activity score; ASQoL: ankylosing spondylitis quality of life; ESR: erythrocyte sedimentation rate; CRP: C-reactive protein; AE: adverse events.

## Data Availability

The datasets used and analyzed during the current study are available from the corresponding author on reasonable request.

## Abbreviations

AS: Ankylosing spondylitis
WZG: Wenbu Zhibi granule
ASAS: Assessment of SpondyloArthritis international society
SPARCC: Spondyloarthritis research consortium of Canada
BASDAI: Bath ankylosing spondylitis disease activity index
BASFI: Bath ankylosing spondylitis functional index
ASDAS: Ankylosing spondylitis disease activity score
BASMI: Bath ankylosing spondylitis metrology index
Asqol: Ankylosing spondylitis quality of life
ESR: Erythrocyte sedimentation rate
CRP: C-reactive protein
NSAIDs: Non-steroidal anti-inflammatory drugs
DMARDs: Disease-modifying anti-rheumatic drugs
TNF: Tumor necrosis factor
IL-17: Interleukin-17
SLE: Systemic lupus erythematosus
RA: Rheumatoid arthritis
HGB: Hemoglobin
PLT: Platelet
ALT: Alanine aminotransferase
AST: Aspartate aminotransferase
BUN: Blood urea nitrogen
CRE: Serum creatinine.

## Additional Points

The trial is expected to begin on May 1, 2021, and we will complete the recruitment on May 1, 2022.
